# Associations among parental feeding styles and children's food intake in families with limited incomes

**DOI:** 10.1186/1479-5868-6-55

**Published:** 2009-08-13

**Authors:** Sharon L Hoerr, Sheryl O Hughes, Jennifer O Fisher, Theresa A Nicklas, Yan Liu, Richard M Shewchuk

**Affiliations:** 1 Department of Food Science and Human Nutrition, Michigan State University, East Lansing, Michigan, USA; 2 Department of Pediatrics, Baylor College of Medicine, USDA/ARS Children's Nutrition Research Center, Houston, Texas, USA; 3 Department of Public Health, Temple University, Center for Obesity Research and Education, Philadelphia, Pennsylvania, USA; 4 Department of Health Services Administration, University of Alabama at Birmingham, 560 Webb Building, 1530 Third Avenue South, Birmingham Alabama, USA

## Abstract

**Background:**

Although general parenting styles and restrictive parental feeding practices have been associated with children's weight status, few studies have examined the association between feeding styles and proximal outcomes such as children's food intake, especially in multi-ethnic families with limited incomes. The purpose of this study was to evaluate the association of parental feeding styles and young children's evening food intake in a multiethnic sample of families in Head Start.

**Methods:**

Participants were 715 Head Start children and their parents from Texas and Alabama representing three ethnic groups: African-American (43%), Hispanic (29%), and White (28%). The Caregivers Feeding Styles Questionnaire (Hughes) was used to characterize authoritative, authoritarian (referent), indulgent or uninvolved feeding styles. Food intake in several food groups was calculated from 3 days of dietary recalls for the child for evening food intakes from 3 PM until bedtime.

**Results:**

Compared to children of authoritarian parents, intakes of fruits, juice and vegetables were lowest among children of indulgent or uninvolved parents (1.77 ± 0.09 vs 1.45 ± 0.09 and 1.42 ± 0.11 cups) as were intakes of dairy foods (0.84 ± 0.05 vs 0.67 ± 0.05 and 0.63+0.06 cups), respectively.

**Conclusion:**

Findings suggest that permissive parent feeding styles like indulgent or uninvolved relate negatively to children's intake of nutrient-rich foods fruit, 100% fruit juice, vegetables and dairy foods from 3 PM until bedtime.

## Background

The interactive behavioral processes occurring between parents and children surrounding eating have become a recognized influence on children's eating behaviors and weight status [1-3]. Unfortunately, the research literature linking parenting behaviors to child eating and weight status has followed two separate and distinct paths resulting in some confusion in the field. One path involves a series of studies (laboratory, cross-sectional, and longitudinal) on a circumscribed set of parental feeding *practices *(restriction, monitoring, and pressure to eat). Parenting practices, by definition, are considered behaviors that parents use to get children to do something specific, in this case to control children's eating. In general, laboratory studies have demonstrated negative effects of high levels of restriction and pressure to eat on aspects of children's self-regulation of energy intake and satiety [4-6]. Moreover, restriction, in particular, a highly controlling feeding practice, has also been consistently associated with overweight and weight gain in children across multiple studies [7-9].

A separate research path has emerged recently in the literature associating general parenting styles with children's overweight status. General parenting *style *is a global and stable characteristic of parenting reflecting both the degree of demands/control on the child as well as the parental responsiveness to the child [10]. Parenting styles are considered distinct from parenting practices, because parenting practices and the meaning of those practices for children's development are embedded in the larger parenting style [9]. In a large survey sample in which general parenting styles were measured, Rhee [11]found child overweight most prevalent in those with authoritarian parents (highly demanding, but not very responsive), a finding consistent with earlier work on high parental control of children's food intake and child self-regulation [12]. In Rhee and other studies, permissive parenting styles, involving high parental responsiveness to the child, but few demands, were also associated with increased risk for child overweight [11,13,14]. These findings on permissive parenting styles, associating low parental demandingness/control with child overweight, contrast with earlier work showing high parental control related to poor eating self-regulation and overweight in children. As such, it is unclear what specific mechanisms in general parenting styles lead to overweight status in children. Unfortunately, this set of literature on general parenting styles lacks the specificity of context (see Costanzo and Woody, 1985 for an overview of context specific parenting)[15] and leaves many possibilities for intervening influences.

More recently, the concept of *feeding *styles has been introduced into the literature which embeds how parents interact with children around eating within a general parenting style framework. Studies using this new conceptualization in which general parenting styles are characterized within the context of child feeding show positive associations between permissive *feeding *styles and children's weight status [16,17]. These findings are similar to those of Rhee [11] and others [13,14] suggesting that too little demandingness may not be adaptive in the current dietary climate. In contrast with studies showing a positive association between children's weight status and authoritarian feeding practices (see Clark, 2007 for review)[7] as well as authoritarian parenting styles [11], Hughes and colleagues found authoritarian *feeding *styles to be negatively related to children's weight status [17,18]. Findings from studies using this new concept of feeding styles suggest that some level of parental demandingness is probably necessary to promote optimal eating and ultimately weight outcomes in young children [3,16,19].

To date, there have been relatively few studies showing associations between parenting/feeding styles and children's food intake [9,20]. Furthermore, a noteworthy qualification of many studies involving parenting styles and feeding practices is that they have been conducted, for the most part, with middle-class White samples [9,21]. Therefore, the aim of this study was to determine if and how feeding styles of parents are related to what children eat, specifically in children from families with limited incomes and of diverse race-ethnicity. The authoritarian feeding style, high in demandingness, was the referent in this study out of interest in integrating the existing literature on authoritarian feeding practices with authoritarian parenting/feeding styles. Based on preliminary work on feeding styles and child weight [17,18], the hypothesis was that children of parents with authoritarian feeding styles would consume higher levels of most fruits, vegetables and dairy foods and lower levels of energy-dense diets as compared to children of parents who were permissive in their feeding styles, such as indulgent and uninvolved.

## Methods

### Sample

Parent-child dyads selected were from a study designed to investigate the facilitators and barriers to fruit and vegetable intake of parent-child dyads in Head Start families recruited from Head Start centers in Alabama and Texas. Selection criteria included being a non-pregnant primary caregiver, having a child enrolled in Head Start in his or her first year of participation and between 3-5 years of age, having an income at or below 100% of the poverty index, and self-identification of race/ethnicity as African-American (AA); Hispanic American (HA) or White (W). The primary caregiver was the person most often responsible for what the Head Start child consumed outside of preschool. Of these caregivers, 95% were female (93% mothers, 6% grandmothers, 1% other) and 5% were male. Because only a few caregivers were not parents; all are referred to as 'parents' through out the remainder of this study. Of those who agreed to be interviewed, 715 parents completed the feeding styles questionnaire and reported dietary data on their children.

### Procedures

Following approval by the Institutional Review Boards from the Baylor College of Medicine and Temple University and in compliance with the Declaration of Helsinki (1996), Head Start personnel sent recruitment flyers to parents about their interest in participation. Parents and children who fit the selection criteria signed consent forms and were interviewed at the Head Start centers during fall 2004 to fall 2005. Bilingual data collectors were matched by race/ethnicity to the parents they interviewed. Demographic data were elicited such as marital status, level of education, and race-ethnicity. The interviewers measured heights and weights and collected three dietary recalls on the parent and their preschool child. A packet of questionnaires including feedings styles was sent home with the parents who returned the completed forms to the Head Start centers in sealed envelopes. Upon data completion, parents received incentives of cash and food coupons.

### Anthropometric measures

Data collectors collected weight and height measurements twice on each parent and child without shoes and dressed in light clothing using standardized protocols [22]. Weight was measured to the closest 0.1 kg on a digital platform scale accurate to 500 kg within ± 0.05 kg. (Befour Model PS-6600, Saukville, WI). Height was measured to the closest 0.1 cm using an adult height measuring board (Shorr Productions Growth Unlimited, Olney, MD). Body Mass Index was calculated (BMI = wt in kg/ht m^2^). Height and weight scores for the children were averaged and converted to age- and gender-specific BMI Z scores using the 2000 growth charts from the Centers of Disease Control and Prevention [23].

### Feeding styles

The self-administered Caregivers Feeding Styles Questionnaire (CFSQ) was used to assess the parental feeding style of parents during the dinner meal [17]. In this typological approach, two scores were derived for demandingness/control and responsiveness/warmth from 12 parent-centered feeding items and 7 child-centered feeding items (response scores were 1 = never to 5 = always). Median splits calculated on these two dimensions into high and low permitted categorization into one of four parenting styles-authoritative, authoritarian, indulgent, and uninvolved-as follows. Because all feeding items assessed the degree to which parents reported doing something to encourage or discourage children's eating behavior, the mean of all 19 items formed the demandingness/control score (a measure of the degree to which parents tried to get their child to eat, regardless of the type of feeding strategy used). To derive the score for responsiveness (a measure of the type of strategy that controlled for the level of demandingness), the mean of the seven child-centered items was divided by the mean of the 19 items for each parent, resulting in a measure of the degree to which the parent used child-centered versus parent-centered techniques for child-eating behaviors. Evidence of test-retest reliability, internal consistency, convergent validity, and predictive validity has been shown with a low-income sample [17].

### Dietary variables

Dietary intakes from three days-one weekend day and two non-sequential weekdays - from 3 PM until bedtime were averaged for the children. Only the dietary intakes of the children from 3 PM in the afternoon until the child's bedtime were used (referred to hereafter as evening foods), because the study focused on the dinner time feeding styles of the parents. The children attended Head Start from 7 AM until 2 PM, so on weekdays their dietary intakes were unavailable for breakfast, lunch and morning snacks. For 42 children only two days of dietary recalls were available and these were averaged. The USDA multiple pass protocol was used to collect the dietary recall data because it is considered a standardized method [24]; two-dimensional food models assisted parents with accuracy in portion size recall [25]. Each parent provided information about the foods and beverages consumed by their children within the previous 24 hours when the children were not at school. Although dietary supplement information was collected, such data were not included because the study focused on the children's food intake. For each food or beverage the mother provided time of consumption, amount ingested, the location of purchase or preparation, and identified each food and beverage occasion as a specific meal. Dietary data were collected and analyzed using Nutrient Data System for Research (version 5.0_35, 2004 developed by the Nutrition Coordinating Center, University of Minnesota, Minneapolis, MN). The NDS-R database contains 18,000 foods including ethnic foods and quantifies nutrient intakes and food group servings.

Mean intakes of foods and beverages of interest were reported as the five main food groups of fruit including 100% fruit juice, vegetables, dairy foods, grains, meats. The food group serving sizes were those in MyPyramid [26], that is 1 cup equivalents for fruit and vegetables; 300 mg calcium equivalents for dairy; 1 oz flour equivalents for grains; 1 oz meat equivalents. Energy density (kcal/g of food/beverage) was calculated three ways dividing the average daily calories consumed after 3 pm by the food weight in grams. The energy density of foods consumed was calculated for: (1) all foods and beverages; (2) all foods and energy containing beverages including milk, juices, sodas, sweet ice tea, juice drinks, etc; and (3) all foods, but not beverages [27]. To date there is no agreement on the best energy density measure to use so all are reported in this study.

### Data Analysis

All statistical analyses conducted with the data were run using Statistical Analysis Software (SAS) version 9.1.3 (SAS Institute Inc, Cary, NC, 2006). The significance level was set to 0.05 for overall analyses. Means and standard deviations as well as frequency distributions of participants' characteristics were generated. Missing data were handled on a case by case basis to maximize the information derived for analysis. Dietary intake variables included 24-hr energy intake (kcal), energy densities (kcal/g), and amount of foods from selected food groups (in servings/per day). Analysis of variance (ANOVA), with a priori specific contrasts (using parents with authoritarian feeding style as the referent) adjusting for the number of contrasts so that family-wise error rate would equal 0.05, was performed to examine differences in dietary intake data between the feeding styles. Thus the three comparisons had to exceed a *P *value of < 0.0167 to attain significance. To examine differences in feeding style classifications for categorical variables (e.g., race) Chi square analyses were conducted. Least square means were obtained by using the SAS procedure GLM, adjusted for the child's BMI Z-score.

## Results

Most parents had feeding styles categorized as authoritarian (30.6%) or indulgent (33.3%) **(Table S1; **Additional File 1). For AA parents the indulgent feeding style predominated and for the HA, the authoritarian feeding style did. There were small, significant differences in the age of the child and the household size by parental feeding style, but these were not clinically meaningful. The average BMI of the parents was within the obese range, ≥ 30 kg/m^2^. The BMI Z scores of these Head Start children were high with children of indulgent parents tending to have the highest BMI Z scores and children of authoritarian parents, the lowest (a priori comparison *P *= 0.056). The standard deviations in the BMI Z scores were large.

The associations of feeding styles with the energy density of evening foods consumed by children are shown in **Table S2; **Additional file 2. Compared to authoritarian feeders, those with uninvolved (*P *= 0.023) or with indulgent feeding styles (*P *= 0.030) had children who consumed evening foods with a higher energy density.

Preschool children of indulgent or uninvolved parents had lower evening intakes of fruits, 100% fruit juices and vegetables compared to those whose parents had authoritarian feeding styles (1.77 ± 0.09 vs. 1.45 ± 0.09 and 1.42 ± 0.11 cups,) as well as the lowest intakes of dairy foods (0.84 ± 0.05 vs. 0.67 ± 0.05 and 0.63 ± 0.06 cups), respectively **(Table S3; **Additional File 3). The average evening intakes of children of authoritative parents were between those of children with authoritarian and permissive (indulgent or uninvolved) feeding styles. Only for grains, did the children of authoritative parents have evening intakes lower than that of authoritarian parents (p < 0.0167).

## Discussion

This study integrated and moved beyond the existing literature on restrictive feeding practices and general parenting styles, by using parental feeding styles with limited income families and by setting authoritarian feeding style as the referent. Results demonstrated that young children of parents with permissive feeding styles (indulgent or uninvolved) had the most energy-dense diets and this study was one of the few to report energy density of young children [28]. Results also showed that children of parents with permissive feeding styles had the lowest evening intakes of fruits, fruit juices, vegetables and dairy foods, as compared to the authoritarian feeding style. Such findings extend the literature on parent-child interactions and children's intake/weight status beyond studies focusing solely on restrictive feeding practices or general parenting styles among predominantly White, middle-class parents [11,12]. It was also important to extend this research to families with limited incomes and diverse race-ethnicities, due to differences in cultural practices and because their children are at a higher risk of becoming overweight compared to those of middle income white families [29]. The present study was unique in examination of the influence of feeding styles (context specific parenting styles) on children's dietary intake to determine if and how feeding styles of low-income parents were related to what children actually ate. A major advantage of the present study was that the selected measures were linked to constructs of interest thus highlighting the specific mechanism (i.e., feeding styles instead of general parenting styles) to the children's food intake that can indirectly affect weight status.

In contrast to studies showing authoritative parenting associated with positive child outcomes[30], our findings demonstrated that an authoritarian feeding style associated with better eating behaviors in low-income children. While authoritarian parenting may not support children's internalization of parental values as described in the general parenting literature, it may be more effective in the eating domain by facilitating moderation of intake in children and consumption of nutrient-rich foods. For example, while the child is young, the parent has greater control of the food environment compared to when the child is older and often eating outside the home [31].

Spurrier and colleagues recently found that parental restriction of access by children to less healthy foods was associated with better dietary patterns while coercive behaviors were associated with poorer ones [32]. Furthermore, among samples of families with limited incomes and those with different cultural backgrounds, authoritarian parenting has been shown to be effective and beneficial. For example, Baldwin, Baldwin, and Cole [33] found that, for African-American youth living in disadvantaged circumstances, authoritarian parenting behaviors were associated with better cognitive, socio-emotional, and health outcomes in children. Similarly, Lamborn, Dornbush, and Steinberg [34] found better adjustment in African-American adolescents compared to Whites when their parents used authoritarian parenting in decision making processes.

Therefore, it might be that within the eating context, parental demandingess or direction is positive especially with young children and those from some cultural and socioeconomic groups. Demandingness was defined in this study as the degree to which parents reported doing something to encourage or discourage children's eating. Therefore, high demandingness in the eating context has to do with the amount of directives used by the parenting during eating. The fact that afternoon and evening food intakes were improved when parents exhibited higher demandingness gives support to the idea that more encouragement (or discouragement) in the feeding context may facilitate better quality intake. Given the current dietary environment with fast food restaurants plentiful and energy dense foods commonly available in shopping malls and movie theatres [35], feeding styles that place demands on children to guide their eating behaviors might be adaptive. Furthermore, feeding styles may be a proxy for other aspects of how parents approach feeding such as organization and structure of the eating environment and availability of foods in the home [31,32]. Having scheduled mealtimes and serving foods that are appetizing and nutrient-rich likely permits the introduction and development of better eating habits in young children [32].

There were several limitations to the present study. Use of a convenience sample, however large, limits the generalizability of findings, and Asian families were not included in the sample. All data were parent-reported, making social desirability a potential issue, even though the questionnaires were completed voluntarily and responses were confidentially coded. Cross-sectional studies cannot address causality, so it remains unclear the degree to which the parental feeding behaviors are an influence or result of children's intake. Furthermore, longitudinal data are needed to address how this type of parental demandingness relates to the development of children's preferences and intake of specific foods over time. Whether this type of demandingness continues to be effective as children age is yet to be determined. While children's intake in the late afternoon and evening may not necessarily represent the full picture of their daily intake, this is the time of day when parents are with their children and, during the dinner meal, interactions are focused specifically on eating. Therefore, later afternoon and evening meals afford a unique opportunity for a closer examination of the influence of parent-child behavioral processes on the specific intake of the child. Intuitively it makes sense to eliminate non-caloric beverages from the calculation of energy density. It might be, however, that parents who served water and/or non-caloric beverages for evening meals and snacks were able to keep energy intakes balanced with energy needs. The significantly higher intakes of beverages by children whose parents exhibited an authoritarian feeding style might support this perspective. Finally, the relationships between feeding styles and the child's food consumption might be confounded by the parent's BMI. However, Wardle found that obese mothers were no more likely than normal weight mothers to practice restrictive feeding with their children, suggesting that "the stereotype of the obese mother who uses food in nonnutritive ways is likely a myth" [36].

## Conclusion and implications

Findings suggest that permissive feeding styles like indulgent or uninvolved were associated with lower children's intakes of nutrient-rich foods like fruit, 100% juice, vegetables and dairy in the afternoon and evening. It is time to move research in this area beyond general parenting style to context specific feeding style and beyond food restriction to the broader feeding context to understand how mealtime feeding behaviors relate to the child's food intake, nutritional and weight status. It seems that restrictive feeding studies may be too limited and the research on general parenting styles and overweight are too broad. We suggest the examination of context specific parenting food practices to know how these influence what children eat and understand how best to intervene.

## Competing interests

The authors declare that they have no competing interests.

## Authors' contributions

SLH, SOH, JOF, TAN, YL, RMS made substantial contributions to the conception, design, acquisition, analysis and interpretation of the data.

SLH, SOH, JOF have been involved in drafting the manuscript or revising it critically for important intellectual content.

SLH, SOH, JOF, TAN, YL, RMS have given final approval of the version to be published.

## Supplementary Material

Additional file 1 **Table S1; Demographics of sample by parental feeding style, mean ± standard deviation (SD) and percentages**. Table showing the Demographics of sample by parental feeding style, mean ± standard deviation (SD) and percentages. ^1 ^Post hoc tests evaluated 3 comparisons between feeding styles where authoritative = 1, authoritarian = 2, indulgent = 3, uninvolved = 4. Significant differences given at p < 0.017 were indicated by the following superscripts - a: 2-3; b: 2-4; c: 2-1. ^2 ^Numbers do not always equal 715, because not all participants provided complete data.^3 ^BMI = wt in kg/ht in m^2^. Click here for file

Additional file 2 **Table S2; Children's energy and energy density intake by parental feeding styles, mean and standard error (SE)**. Table showing Children's energy and energy density intake by parental feeding styles, mean and standard error (SE). ^1 ^Adjusted for BMI Z score. ^2 ^Post hoc tests evaluated 3 comparisons between feeding styles where authoritative = 1, authoritarian = 2, indulgent = 3, uninvolved = 4. Significant differences given at p < 0.017 were indicated by the following superscripts - a: 2-3; b: 2-4; c: 2-1. ^3 ^Energy density A = all foods and all beverages for evening foods; (2-4 p = 0.023). ^4 ^Energy density B = all foods and energy containing beverages (i.e., no water, diet sodas, unsweetened tea) for evening foods; (2-3: p = 0.030). ^5 ^Energy density C = all foods (no beverages) for evening foods. Click here for file

Additional file 3 **Table S3; Children's food group intake^1 ^for evening foods by parental feeding styles, mean and standard error (SE).^2^**. Table showing children's food group intake^1 ^for evening foods by parental feeding styles, mean and standard error (SE).^1^Adjusted for BMI Z score. ^2^Post hoc tests evaluated 3 comparisons between feeding styles where authoritative = 1, authoritarian = 2, indulgent = 3, uninvolved = 4. Significant differences given at p < 0.017 were indicated by the following superscripts - a: 2-3; b: 2-4; c: 2-1. Click here for file
