# Supplementary material for: Associations among parental feeding styles and children's food intake in families with limited incomes
Source: Int J Behav Nutr Phys Act. 2009 Aug 13;6:55. doi: 10.1186/1479-5868-6-55 (PMC2739505; doi:10.1186/1479-5868-6-55)
Supplement: Additional file 1 — Table S1; Demographics of sample by parental feeding style, mean ± standard deviation (SD) and percentages. Table showing the Demographics of sample by parental feeding style, mean ± standard deviation (SD) and percentages. 1 Post hoc tests evaluated 3 comparisons between feeding styles where authoritative = 1, authoritarian = 2, indulgent = 3, uninvolved = 4. Significant differences given at p < 0.017 were indicated by the following superscripts - a: 2-3; b: 2-4; c: 2-1. 2 Numbers do not always equal 715, because not all participants provided complete data.3 BMI = wt in kg/ht in m2. [file 1479-5868-6-55-S1.doc]

| **Table 1. Demographics of sample by parental feeding style, mean ± standard deviation (SD)** **and percentages^1^.** | | | | | | | | | | | | | | |
| --- | --- | --- | --- | --- | --- | --- | --- | --- | --- | --- | --- | --- | --- | --- |
| **Characteristic^2^** | **Total Sample**  **n=715** | |  | **Authoritative**  **n=117** | |  | **Authoritarian**  **n=219** | |  | **Indulgent**  **n=238** | |  | **Uninvolved**  **n=141** | |
|  | **Mean ± SD** | |  | **Mean ± SD** | |  | **Mean ± SD** | |  | **Mean ± SD** | |  | **Mean ± SD** | |
| **Child Age, yr** (n=714) **^a^** | 4.45 ± 0.63 | |  | 4.47 ± 0.60 | |  | 4.35± 0.64 | |  | 4.53± 0.62 | |  | 4.45 ± 0.61 | |
| **Child BMI Z score** (n=697) | 0.82 ± 1.56 | |  | 0.79 ± 1.54 | |  | 0.70± 1.29 | |  | 0.98± 1.54 | |  | 0.76 ±1.93 | |
| **Household number** (n=715; range) **^c^** | 4.5 ±1.6(1-13) | |  | 4.04±1.3 (2-11) | |  | 4.63±1.7(2-12) | |  | 4.47±1.6 (1-12) | |  | 4.48±1.7 (2-13) | |
| **Caregiver Age, yr** (n=714) | 31.60 ± 8.18 | |  | 31.87 ± 9.21 | |  | 31.29± 7.45 | |  | 32.03± 8.39 | |  | 31.10 ±8.04 | |
| **Caregiver BMI^3^ (**n=692) **^a^** | 31.34 ± 8.13 | |  | 31.35 ± 8.04 | |  | 30.20 ± 7.39 | |  | 32.11 ± 8.40 | |  | 31.79 ± 8.72 | |
|  | **n** | **%** |  | **n** | **%** |  | **n** | **%** |  | **n** | **%** |  | **n** | **%** |
| **Child overweight,**  BMI Z score >95^th^ percentile  **percentil** | 173 | 25 |  | 25 | 14 |  | 45 | 26 |  | 69 | 40 |  | 34 | 20 |
| **Caregiver obesity,** BMI >30 | 340 | 49 |  | 52 | 15 |  | 95 | 28 |  | 125 | 37 |  | 68 | 20 |
| **Race/Ethnicity**, **self defined** |  |  |  |  |  |  |  |  |  |  |  |  |  |  |
| African American **^a,c^**  H | 309 | 43 |  | 39 bmpcare.mea729_debra_ed; bmpcare.mea729_debra_ed; | 13 |  | 78 | 25 |  | 125 | 40 |  | 67 | 22 |
| Hispanic **^b,c^** | 207 | 29 |  | 34 | 16 |  | 82 | 40 |  | 55 | 27 |  | 36 | 17 |
| White | 199 | 28 |  | 44 | 22 |  | 59 | 30 |  | 58 | 29 |  | 38 | 19 |
| **Education completed** | | | | | | | | | | | | | | |
| Less and some High School **^b,c^** | 182 | 26 |  | 25 | 14 |  | 71 | 39 |  | 52 | 28 |  | 34 | 19 |
| High School | 239 | 33 |  | 46 | 19 |  | 70 | 29 |  | 74 | 31 |  | 49 | 21 |
| Some College/Technical **^b,c^** | 231 | 32 |  | 34 | 15 |  | 62 | 27 |  | 94 | 40 |  | 41 | 18 |
| College Grad and Higher | 63 | 9 |  | 12 | 19 |  | 16 | 25 |  | 18 | 29 |  | 17 | 27 |
|  | **n** | **%** |  | **n** | **%** |  | **n** | **%** |  | **n** | **%** |  | **n** | **%** |
| **Marital Status** | | | | | | | | | | | | | | |
| Married **^b,c^** | 341 | 48 |  | 65 | 19 |  | 111 | 32 |  | 98 | 29 |  | 67 | 20 |
| Divorced/Widowed/Separated **^c^** | 145 | 20 |  | 21 | 14 |  | 43 | 30 |  | 56 | 39 |  | 25 | 17 |
| Never Married | 182 | 25 |  | 25 | 14 |  | 47 | 26 |  | 70 | 38 |  | 40 | 22 |
| Other | 47 | 7 |  | 6 | 13 |  | 18 | 38 |  | 14 | 28 |  | 9 | 19 |
| **Relationship** |  |  |  |  |  |  |  |  |  |  |  |  |  |  |
| Parent | 629 | 93 |  | 102 | 16 |  | 197 | 31 |  | 203 | 32 |  | 127 | 21 |
| Grandparent | 43 | 6 |  | 6 | 14 |  | 11 | 26 |  | 20 | 46 |  | 6 | 14 |
| Aunt/Uncle | 2 | 0.7 |  | 1 | 20 |  | 2 | 40 |  | 2 | 40 |  | 0 | 0 |
| Other | 2 | 0.3 |  | 0 | 0 |  | 1 | 50 |  | 0 | 0 |  | 1 | 50 |

^1^ Post hoc tests evaluated 3 comparisons between feeding styles where authoritative = 1, authoritarian = 2, indulgent = 3, uninvolved = 4. Significant differences given at p <0.017 were indicated by the following superscripts - a: 2-3; b: 2-4; c: 2-1.

^2^ Numbers do not always equal 715, because not all participants provided complete data.

^3^ BMI = wt in kg/ht in m^2^.
