# Supplementary material for: Associations among parental feeding styles and children's food intake in families with limited incomes
Source: Int J Behav Nutr Phys Act. 2009 Aug 13;6:55. doi: 10.1186/1479-5868-6-55 (PMC2739505; doi:10.1186/1479-5868-6-55)
Supplement: Additional file 2 — Table S2; Children's energy and energy density intake by parental feeding styles, mean and standard error (SE). Table showing Children's energy and energy density intake by parental feeding styles, mean and standard error (SE). 1 Adjusted for BMI Z score. 2 Post hoc tests evaluated 3 comparisons between feeding styles where authoritative = 1, authoritarian = 2, indulgent = 3, uninvolved = 4. Significant differences given at p < 0.017 were indicated by the following superscripts - a: 2-3; b: 2-4; c: 2-1. 3 Energy density A = all foods and all beverages for evening foods; (2-4 p = 0.023). 4 Energy density B = all foods and energy containing beverages (i.e., no water, diet sodas, unsweetened tea) for evening foods; (2-3: p = 0.030). 5 Energy density C = all foods (no beverages) for evening foods. [file 1479-5868-6-55-S2.doc]

| T**able 2. Children’s energy and energy density intake^1^ by parental feeding styles, mean and standard error (SE).^2^** | | | | | |
| --- | --- | --- | --- | --- | --- |
| **Food** | **Total**  **(n=715** ) | **1**  **Authoritative (n=117)** | **2**  **Authoritarian (n=219)** | **3**  **Indulgent (n=238)** | **4**  **Uninvolved (n=141)** |
| **Energy for evening foods** | 646 ± 12.1 | 635 ± 28.6 | 690 ± 20.9 | 645 ± 20.0 | 612 ± 26.2 |
| **Energy Density A**^3^ | 1.11 ± 0.02 | 1.11 ± 0.03 | 1.07 ± 0.02 | 1.12 ± 0.02 | 1.15 ± 0.03 |
| **Energy Density B** ^4^ | 1.24 ± 0.01 | 1.23 ± 0.03 | 1.20 ± 0.02 | 1.28 ± 0.02 | 1.25 ± 0.03 |
| **Energy Density C** ^5^ | 1.76± 0.02 | 1.72 ± 0.04 | 1.77 ± 0.04 | 1.78± 0.03 | 1.77 ± 0.04 |
| ^1^ Adjusted for BMI Z score  ^2^ Post hoc tests evaluated 3 comparisons between feeding styles where authoritative = 1, authoritarian = 2, indulgent = 3, uninvolved = 4. Significant differences given at p <0.017 were indicated by the following superscripts - a: 2-3; b: 2-4; c: 2-1.  ^3^ Energy density A = all foods and all beverages for evening foods; (2-4 p=0.023)  ^4^ Energy density B = all foods and energy containing beverages (i.e., no water, diet sodas, unsweetened tea) for evening foods; (2-3: p=0.030)  ^5^ Energy density C = all foods (no beverages) for evening foods. | | | | | |
