# Supplementary material for: Associations among parental feeding styles and children's food intake in families with limited incomes
Source: Int J Behav Nutr Phys Act. 2009 Aug 13;6:55. doi: 10.1186/1479-5868-6-55 (PMC2739505; doi:10.1186/1479-5868-6-55)
Supplement: Additional file 3 — Table S3; Children's food group intake1 for evening foods by parental feeding styles, mean and standard error (SE).2. Table showing children's food group intake1 for evening foods by parental feeding styles, mean and standard error (SE).1Adjusted for BMI Z score. 2Post hoc tests evaluated 3 comparisons between feeding styles where authoritative = 1, authoritarian = 2, indulgent = 3, uninvolved = 4. Significant differences given at p < 0.017 were indicated by the following superscripts - a: 2-3; b: 2-4; c: 2-1. [file 1479-5868-6-55-S3.doc]

| **Table 3. Children’s food group intake^1^ for evening foods by parental feeding styles, mean and standard error (SE).^2^** | | | | | |
| --- | --- | --- | --- | --- | --- |
| **Food** | **Total**  **(n=715** ) | **1**  **Authoritative (n=117)** | **2**  **Authoritarian (n=219)** | **3**  **Indulgent (n=238)** | **4**  **Uninvolved (n=141)** |
| **Beverage ^a,b^** | 1.52 ± 0.04 | 1.49 ± 0.11 | 1.80 ± 0.08 | 1.39 ± 0.07 | 1.41 ± 0.10 |
| **Dairy ^a,b^** | 0.71 ± 0.03 | 0.69 ± 0.07 | 0.84 ± 0.05 | 0.67 ± 0.05 | 0.63 ± 0.06 |
| **Grain ^c^** | 2.08 ± 0.05 | 1.82 ± 0.13 | 2.27 ± 0.09 | 2.15 ± 0.09 | 2.07 ± 0.12 |
| **Meat** | 1.80 ± 0.04 | 1.89 ± 0.11 | 1.73 ± 0.08 | 1.92 ± 0.07 | 1.64 ± 0.10 |
| **Fruit, 100% Juice,**  **Vegetables ^a,b^** | 1.56 ± 0.05 | 1.59 ± 0.12 | 1.77 ± 0.09 | 1.45 ± 0.09 | 1.42 ± 0.11 |
| ^1^Adjusted for BMI Z score  ^2^Post hoc tests evaluated 3 comparisons between feeding styles where authoritative = 1, authoritarian = 2, indulgent = 3, uninvolved = 4. Significant differences given at p <0.017 were indicated by the following superscripts - a: 2-3; b: 2-4; c: 2-1. | | | | | |
|  | | | | | |
